# Challenges in Residual Bearing Removal: A Rare Case of Mobile Bearing Fracture in Unicompartmental Knee Arthroplasty with Literature Review

**DOI:** 10.1111/os.14138

**Published:** 2024-07-01

**Authors:** Xianyue Shen, Xianzuo Zhang, Yang Liu, Chen Zhu, Wei Huang

**Affiliations:** ^1^ Department of Orthopedics, The First Affiliated Hospital of USTC, Division of Life Sciences and Medicine University of Science and Technology of China Hefei China

**Keywords:** Bearing fracture, Case report, Mobile bearing, Unicompartmental knee arthroplasty

## Abstract

**Background:**

Mobile bearing fracture is a rare long‐term complication of unicompartmental knee arthroplasty (UKA), and relevant reports are sparse. Hence, its treatment options need further exploration.

**Case Presentation:**

This study presents the case of fracture of a polyethylene insert that occurred 12 years after mobile bearing medial UKA in a 75‐year‐old overweight woman who then underwent surgical intervention at our institution. However, we encountered significant challenges in removing the fragments from the broken bearing, resulting in retention of the remaining one‐third of the fragment. We solved this problem by replacing the fractured insert with thicker mobile bearing. During the 1‐month postoperative follow‐up, the patient achieved good range of motion and excellent satisfaction, with no reported complications and a Knee Society Score of 90. Additionally, we reviewed the literature on the treatment for mobile bearing fractures after UKA.

**Conclusions:**

Bearing fracture is a rare cause of failure of mobile bearing UKA. This case highlights the challenges of UKA fracture bearing retrieval and underscores that mobile bearing replacement can be an effective intervention. The case we report shows that when removal of a residual meniscal bearing in a posterior dislocation is difficult to achieve, compromise may be an appropriate option because it does not cause additional complaints to the patient. This case emphasizes the importance of the surgeon having a thorough preoperative understanding of the location and potential pitfalls of fracture fragments in such situations.

## Graphical abstract

In this work, we introduced a 75‐year‐old female patient who developed mobile bearing fracture 12 years after UKA surgery and received surgical intervention at our institution. However, we encountered a significant challenge in removing fragments from the broken bearing, resulting in the remaining one‐third of the fragment being unable to be removed. But we resolved this issue by replacing a thicker mobile bearings to compromise the remaining fragments, and the patient achieved good range of motion and satisfaction. Our work emphasizes the importance of the surgeon having a thorough preoperative understanding of the location and potential pitfalls of fracture fragments in such situations.

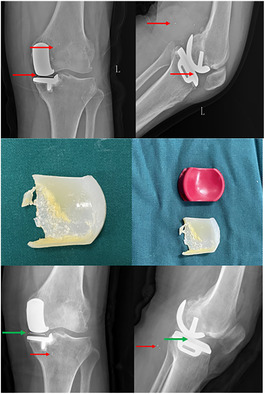

## Introduction

Unicompartmental knee arthroplasty (UKA) has emerged as an effective surgical intervention for patients with end‐stage unicompartmental knee osteoarthritis.^1^ UKA has significantly improved and rapidly grown over the past two decades, owing to advancements in surgical and prosthetic techniques. Among them, the Oxford mobile bearing UKA is the most widely used. The polyethylene insert it uses functionally mimics the natural meniscus, making it fully consistent throughout the entire knee range of motion, reducing contact stress, and theoretically reducing polyethylene insert wear. Currently, mobile bearing UKA has demonstrated reliable long‐term clinical results. A multicenter study showed that the 10‐year prosthesis survival rate in mobile bearing UKA (Oxford) can reach 90%, showing that mobile bearing UKA has an excellent mid‐term prosthesis survival rate.^2^ Additionally, some research evidence supports that UKA offers more natural knee motion function, increased range of motion, reduced complications and mortality, faster recovery, and shorter hospitalization than total knee arthroplasty (TKA).^3^ Studies have reported that patients undergoing UKA have a 96% chance of returning to preoperative activity levels,^4^ with 90% experiencing improved motion^5^; whereas, the dissatisfaction rate for TKA alone ranges from14% to 19%.^6^

Despite its success, UKA is not without complications, and rare adverse events can occur, posing unique challenges for orthopaedic surgeons.^7^ Previous studies have confirmed that there is no significant difference in long‐term clinical outcomes and complications between mobile and fixed bearing UKA.^8^, ^9^ Although mobile bearing UKA has the biomechanical advantage of achieving near‐normal knee kinematics, it also carries the risk of meniscal bearing dislocation or fracture, a complication unique to this implant design. The risk factors leading to mobile bearing UKA failure and affect implant survival remain controversial. Currently, UKA postoperative failure and revision mainly include periprosthetic infection, bearing dislocation, aseptic loosening, and periprosthetic fracture.^10^ This is usually related to the selection of surgical indications, poor implant position, imbalance of flexion and extension gaps, and other factors. However, meniscal bearing fractures are rare in mobile bearing UKA, as it can result in knee instability and failure, and there are only few related reports.^11^, ^12^, ^13^ This is usually because of long‐term wear of the bearing, use of thin polyethylene bearing for the initial surgery, and postoperative traumatic overloading.

Here, we present a rare case of mobile bearing fracture that occurred 12 years postoperatively in a patient who underwent UKA. The fracture led to considerable challenges in retrieving the remaining bearing during the surgical procedure. We only exchanged the 4 mm mobile bearing, and the remaining bearing was retained in a compromise. This novel approach ultimately allowed the patient to achieve good knee function and pain relief. The aim of this case is to describe our treatment options for difficult‐to‐retrieve residual bearing in the setting of this rare complication. With the experience of this case report, we wish to describe the treatment options for difficult‐to‐retrieve residual bearing in the context of this rare complication. We have also conducted a comprehensive literature review of similar cases to deepen our understanding of this rare complication and explore potential management strategies.

## Case Presentation

A 75‐year‐old woman (weight: 66 kg, height: 152 cm, BMI: 28.5 kg/m^2^) was admitted to our orthopaedic outpatient clinic on February 5, 2024, with complaints of left knee swelling and pain persisting for over 2 months, but no locking symptoms. The patient had a history of symptomatic medial compartment osteoarthritis of the left knee 12 years earlier and subsequently underwent minimally invasive Oxford mobile bearing UKA (Biomet UK Ltd., Bridgend, UK). Postoperatively, the patient experienced pain relief and achieved a satisfactory range of motion in the knee joint. Two months before the visit to our department, the patient reported a sudden burst sensation while walking, followed by subsequent knee pain during ambulation, although she remained asymptomatic during light‐to‐moderate physical activities. The patient had medical history of hypertension (American Society of Anesthesiologists physical status classification II due to hypertension), and was obese (according to the Chinese BMI classification standards), and had undergone abdominal surgery in 2007 (details unspecified). Based on her medical history and imaging findings, we diagnosed left knee bearing fracture.

**FIGURE 1:**
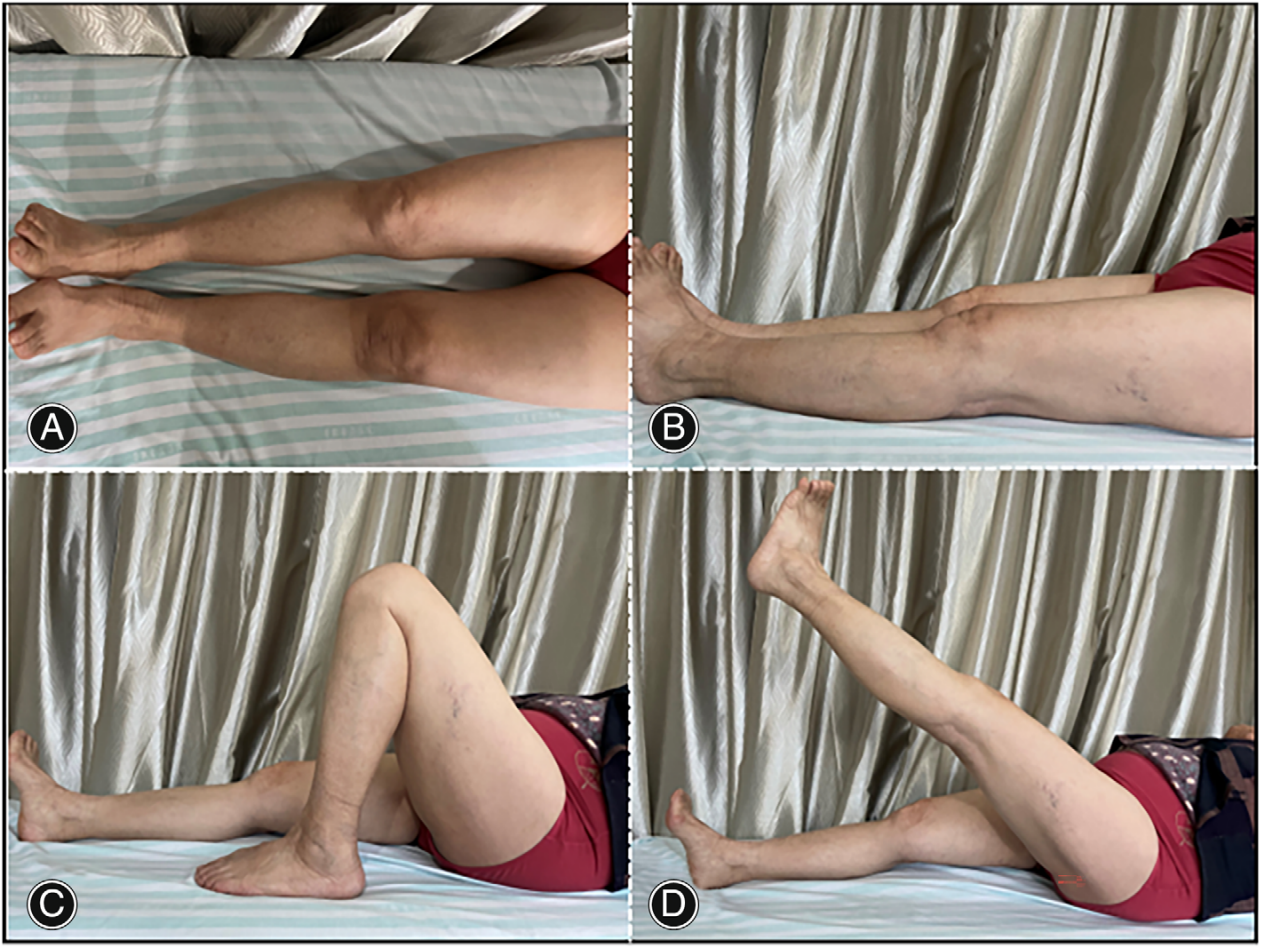
Physical examination indicated satisfactory left knee range of motion. Top view (A); lateral views (B) and (D) show knee extension; and lateral view (C) shows flexion of the left knee.

Upon admission, the patient underwent a comprehensive physical examination. No evident abnormalities in lower limb alignment or any obvious tenderness in the left knee were observed. The range of motion of the left knee was 0°–120° (Figure 1). Significant instability was observed during varus and valgus stress tests at full extension and 90° flexion. No notable abnormalities were found in neurovascular status. The Knee Society Score (KSS) was 75.

**FIGURE 2:**
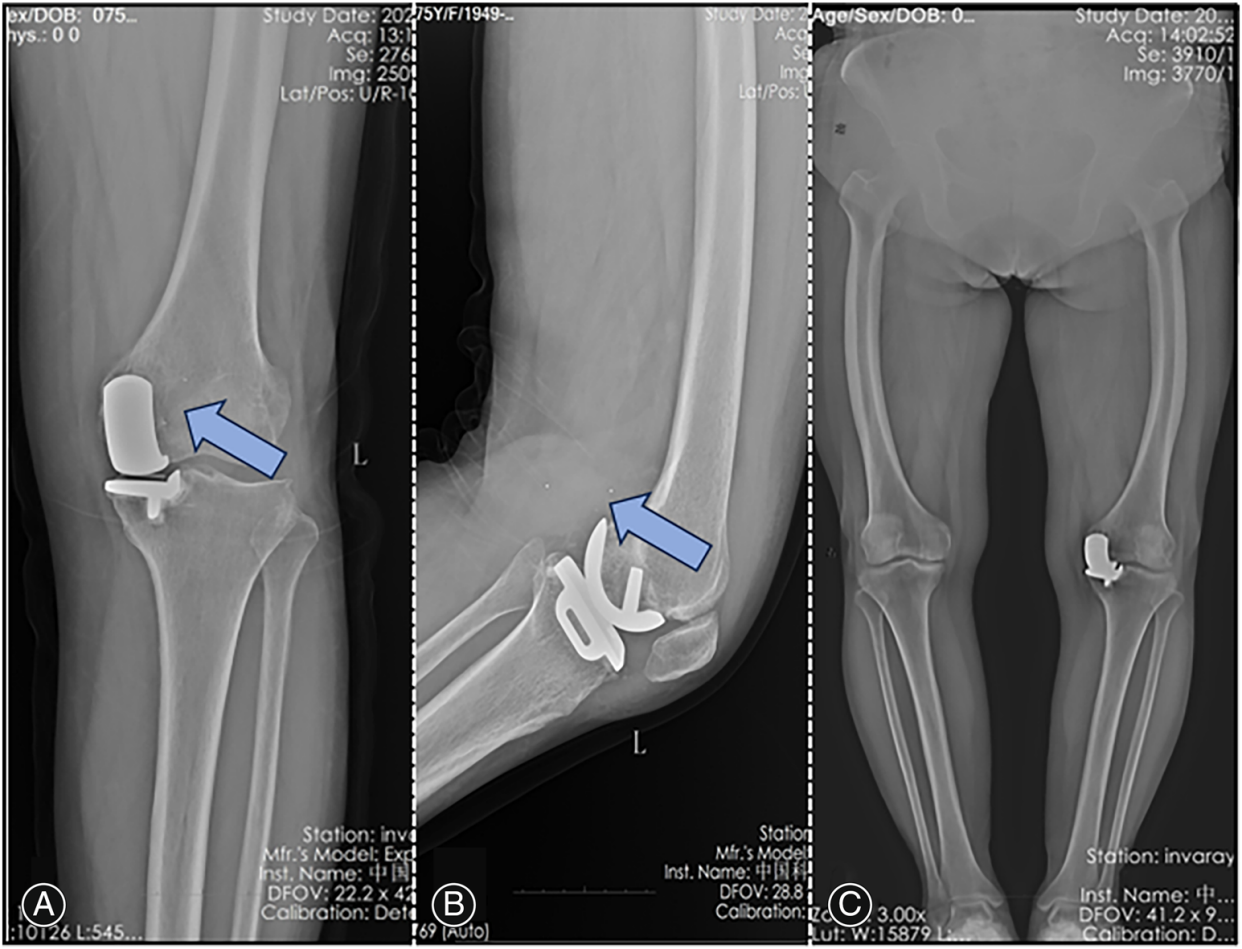
Preoperative radiographs demonstrating that medial unicompartmental knee arthroplasty with mobile bearing fracture and posteromedial dislocation. (A) AP, (B) lateral, and (C) full‐length weight‐bearing of the lower extremities. Blue arrows indicate broken bearing fragments.

Anteroposterior and lateral radiographic views of the left knee and standard full‐length weight‐bearing of the lower extremities revealed evidence of prior medial UKA in the left knee. No apparent loosening of the femoral and tibial components was observed. However, the polyethylene bearing was broken into two fragments and displaced (Figure 2). Laboratory examination revealed normal blood routine, with an erythrocyte sedimentation rate of 25.0 mm/h, and a C‐reactive protein level of 3.08 mg/L, all within the normal range.

**FIGURE 3:**
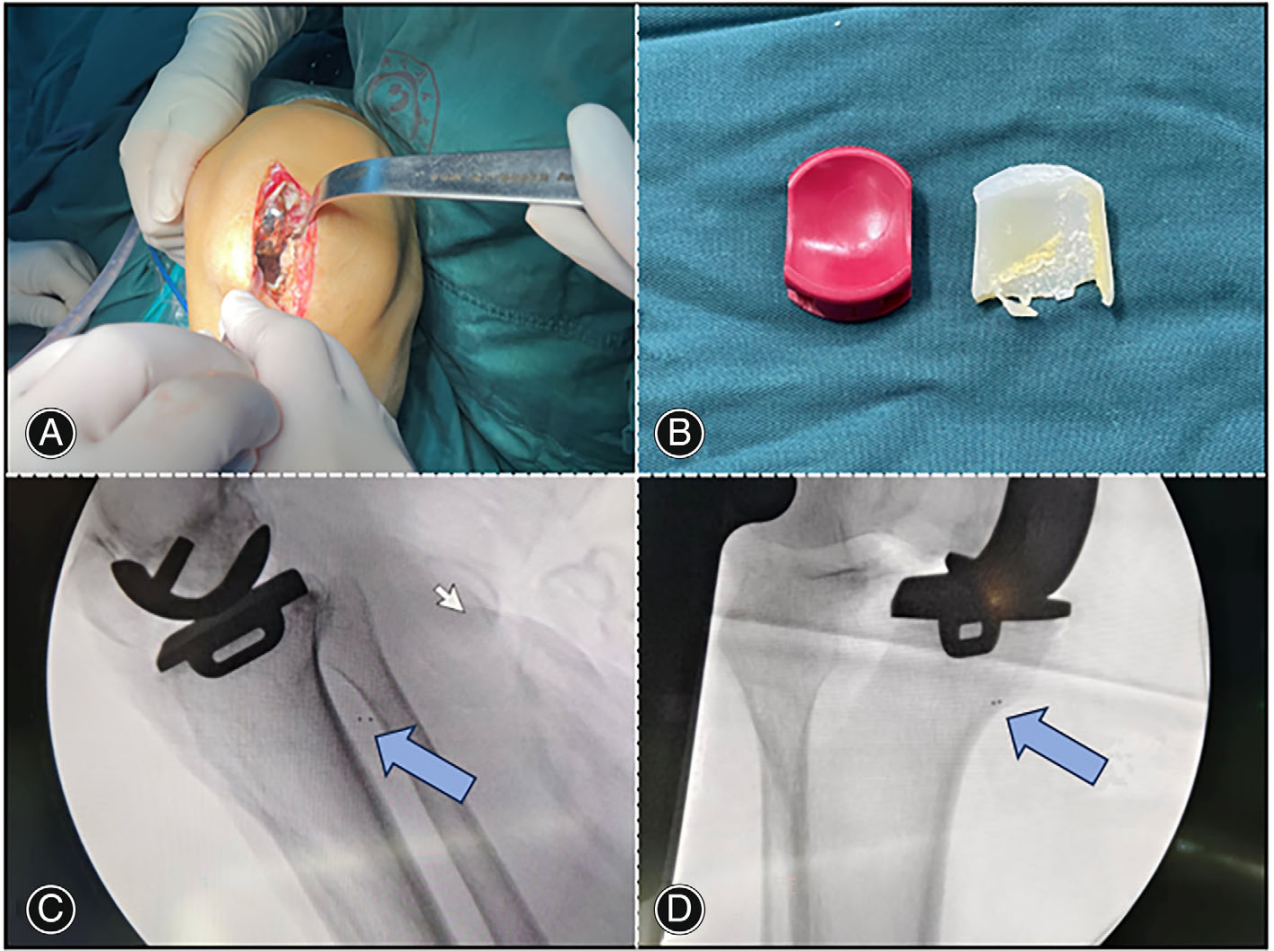
Intraoperative images and fluoroscopy. (A) Shows the knee from the medial incision. (B) Shows the anterior two‐thirds of the removed mobile bearing. (C‐D) Illustrate intraoperative fluoroscopy revealing migration of one‐third of the residual bearing fragments to the posteromedial tibial compartment. Blue arrows indicate broken bearing fragments.

Following a comprehensive review of her medical history, imaging, and laboratory findings, an in‐depth discussion regarding surgical treatment options was carried out with the patient. Our plan was to remove the fractured bearing fragments and replace them with a compatible new mobile bearing. The revision surgery used the previous medial parapatellar approach. Upon exposure of the knee joint, the ruptured fragment of the medial compartment (two‐thirds of the mobile bearing) was removed (Figure 3A,B). Intraoperatively, both the femoral and tibial components were found to be well fixed. Based on preoperative imaging, we determined that the remaining mobile bearing fragment was primarily situated in the posteromedial aspect of the medial femoral condyle. Therefore, we prepared to retrieve it posteriorly through the intercondylar fossa. However, despite proper release, passive knee flexion and extension, and repeated attempts, we were unable to retrieve it. In view of the metal marker on the remaining fragment, we tried to accurately locate it through X‐ray fluoroscopy. Subsequently, intraoperative fluoroscopy revealed the unexpected migration of the remaining fragment from the femoral to the tibial side, where it was situated posteromedially on the tibia (Figure 3C,D). We attempted to locate the fragment from the posteromedial aspect of the tibia, but were still unable to retrieve it. Ultimately, following consultation with the patient's family during the procedure, we opted to forgo further attempts to retrieve the remaining fragments and proceeded only with exchanging the 4 mm thick meniscal bearing. Intraoperative testing showed good range of motion and stability of the knee, along with a favorable bearing motion trajectory.

**FIGURE 4:**
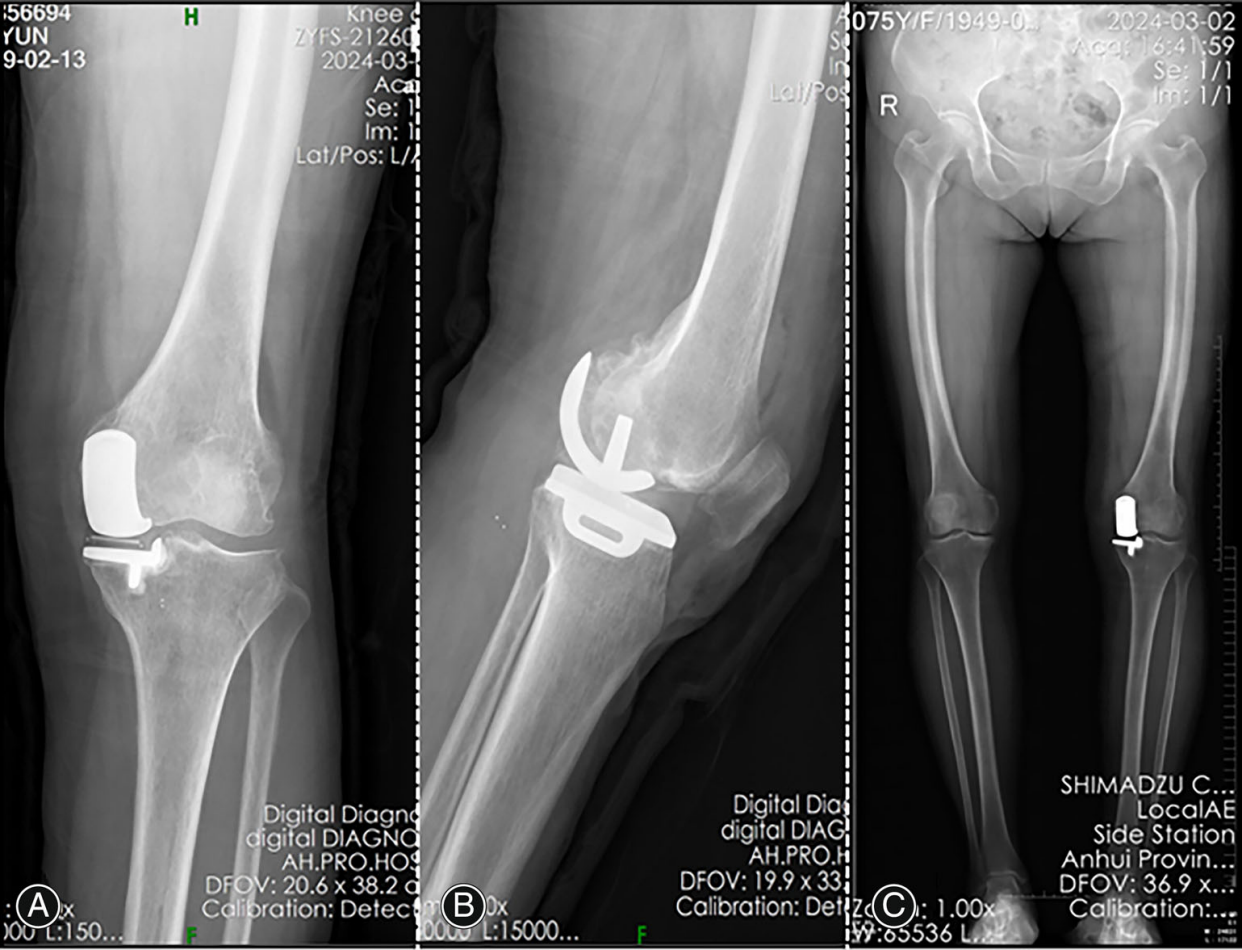
Postoperative radiographs showing replacement of the broken bearing with a 4‐mm thick mobile bearing, with the residual bearing fragment remaining in the left knee. (A) AP, (B) lateral, and (C) full‐length weight‐bearing of the lower extremities.

The day after surgery, the patient was able to walk without assistance and underwent radiographic evaluations (Figure 4). During the 1‐month follow‐up, the incision had healed well. The patient experienced no left knee instability or pain, achieved a KSS clinical score of 90, and reported no further complications.

## Discussion

The case described in this study illustrates the rare occurrence of mobile‐bearing fracture 12 years after UKA and its innovative yet compromising resolution. This unusual complication is often attributed to prolonged wear following the operation. Moreover, the migration of bearing fragments presents significant challenges for surgeons during retrieval, underscoring the importance of thorough preoperative planning.

### Comparison with Other Studies

Previous research suggests that properly executed UKA generally yields extremely low wear rates, typically ranging from 0.01 to 0.02 mm per year.^14^, ^15^ Nonetheless, malalignment or impingement signs can result in abnormally increased wear. Prolonged wear reduces the thickness of the bearing, increasing the stress and predisposing it to fracture in the thinnest regions.^14^ In our case, the mobile bearing fractured during the primary UKA had a thickness of 3 mm, and excessive wear resulted in its fracture in the posterior third. During UKA revision surgery, we performed trial reductions using polyethylene inserts of different sizes to evaluate knee stability and impingement. The final use of a 4 mm meniscus bearing was able to achieve knee stability without varus or valgus, and the meniscus bearing did not lift off. This management strategy was consistent with previous reports.^11^, ^12^ We conducted a comprehensive literature review to elucidate similar cases and to gain insights into optimal management strategies (Table 1). Lim et al.^11^ reported a case of an Oxford UKA, wherein, 7 years after surgery, the patient experienced a bearing fracture attributed to uneven polyethylene delamination and fatigue crack propagation. This complication was effectively managed by replacing the bearing with a thicker one. Munjal^12^ documented a case of bearing fracture after 7 years of treatment with UKA due to oxidative weakening of the thin bearing in a morbidly obese patient. This was resolved by replacing it with an identical‐sized bearing. Additionally, Vajapey et al.^16^ reported a case of mobile bearing fracture 10 years after UKA, which was successfully treated with TKA.

**TABLE 1:** Details of published literature describing mobile bearing fractures after unicompartmental knee arthroplasty.

Year	Reference	Demographic information	Primary UKA bearing details	Time of bearing fracture	Treatment	Outcome
2014	Lim et al.^11^	72‐year‐old female with BMI 23.4 kg/m^2^	Extra small size, 4 mm thick bearing	7 years after UKA	Replaced by a 6 mm‐thick bearing	Good
2018	Munjal^12^	60‐year‐old female	Medium size, 3 mm‐thick bearing	7 years after UKA	Replaced by a same size bearing	Good
2021	Vajapey et al.^16^	40‐year‐old female with BMI 32.28 kg/m^2^	3 mm‐thick bearing	10 years after UKA	Revision to TKA	Good
2023	Ventura et al.^13^	50‐year‐old male, unknown BMI	Medium size, 3 mm‐thick bearing	2 years after UKA	Replaced by a same size bearing	Good

Abbreviations: BMI, body mass index; UKA, unicompartmental knee arthroplasty.

### Review of Retrieval Strategies for Fractured Bearings

Despite limited clinical case reports,^11^, ^12^ no study has yet documented intraoperative challenges in retrieving mobile bearing fragments, a crucial aspect to emphasize. The main dilemma in managing this case was the removal of residual bearings. To our knowledge, this is the first reported case of complex bearing removal in such a rare scenario, highlighting the potential risks and surgical complexities linked to post‐UKA mobile bearing fractures. Fractured polyethylene fragments typically require meticulous retrieval to prevent damage to surrounding tissues and structures. However, because of the minimally invasive nature of UKA incisions, limited access hinders extensive tissue dissection, complicating the identification and removal of bearing fragments. Several methods have been reported for successful retrieval of dislocated mobile bearings, such as the use of extended incisions and arthroscopically assisted techniques.^12^, ^13^ Interestingly, Tibrewal et al.^17^ reported two cases of posterior dislocation of the intact mobile bearing in 2014. They also experienced non‐retrieval of the mobile bearing intraoperatively, which they did by leaving it in situ and simply replacing the bearing. This was similar to our option and both achieved good results. This suggests that compromise may be a safe solution. However, it is understandable that retrieving an intact polyethylene bearing is usually easier than retrieving a broken bearing, considering the ease of exploration. These points further highlight the complexity of UKA revision surgeries. Sharing relevant clinical experiences aims to enhance surgeons' understanding of bearing fractures, alerting surgeons to intraoperative challenges, and ultimately improving clinical outcomes.

Although improvements in implant design, materials, and surgical techniques enhance the longevity of UKA implants and patient outcomes,^18^ late complications like bearing fractures warrant attention, particularly in obese individuals and those with the smallest‐sized inserts. In the event of this complication, effective treatment options such as bearing replacement and TKA are available, usually based on intraoperative evaluation of the femoral and tibial components.

## Conclusion

In summary, this case underscores the challenges of broken bearing retrieval in UKA for the first time, highlighting mobile bearing replacement as an effective treatment intervention. This case emphasizes the importance of surgeons having a comprehensive preoperative understanding of the location and potential pitfalls of fractured fragments in such scenarios. Careful surgical planning and execution are essential to achieve optimal outcomes.

## Conflict of Interest Statement

We declare that we have no conflict of interest.

## Ethics Statement

Patient information provided in this study is anonymous. This study was approved by the Ethics Committee of The First Affiliated Hospital of USTC (IRB NO:2024‐RE‐182).

## Author Contributions

Concept and design: WH and CZ; Acquisition, analysis, or interpretation of data: XYS and YL; Drafting of the manuscript: XYS and XZZ; Critical revision of the manuscript for important intellectual content: WH and CZ; Supervision: WH and CZ.

## Funding Information

The author(s) received no financial support for the research, authorship, and/or publication of this article.

## Consent for Publication

Written informed consent was obtained from the patients for the publication of this study and accompanying images.
